# The study on the complete mitochondrial genome of *Acanthopsetta nadeshnyi* and its phylogenetic position

**DOI:** 10.1080/23802359.2023.2241670

**Published:** 2023-08-10

**Authors:** Jun Young Chae, JinHo Kim, Tae-Wook Kang, Jinkoo Kim, Hyung-Ho Lee, Moo-Sang Kim

**Affiliations:** a Department of Bioinformatics, the MOAGEN, Daejeon, South Korea; b Department of Biotechnology, Pukyong National University, Busan, South Korea; c Major of Marine Biology, Pukyong National University, Busan, South Korea

**Keywords:** Acanthopsetta nadeshnyi, mitochondrial genome

## Abstract

Pleuronectidae is a well-studied familyin the order Pleuronectiformes. In contrast, genetic research on the flatfish *Acanthopsetta nadeshnyi* of the Pleuronectidae family is limited. This study reports the complete mitogenome of *A. nadeshnyi*. The mitogenome was 17,206 bases long and included 13 protein-coding genes (PCGs), 22 transfer RNA (tRNA) genes, two ribosomal RNA (rRNA) genes, and a putative control region. Phylogenetic analysis based on the nucleotide sequences of the 13 PCGs confirmed that *A. nadeshnyi* belongs to the Pleuronectidae family.

## Introduction

The family Pleuronectidae (order Pleuronectiformes) is well-known. Several studies have revealed 23 genera and 61 species in the faily (Nelson et al. 2016). Traditionally, all known flatfish were assigned to the family Pleuronectidae (Jordan and Goss 1889), but were later divided into five families (Norman 1934). *Acanthopsetta nadeshnyi* (Schmidt 1904) is a Pleuronectidae flatfish that lives at depths from 18 to 900 m. It occurs from the Bering Sea and the Sea of Okhotsk to the waters of Japan and Korea (Masuda et al. 1984; Matarese et al. 1987).

The mitochondrial genome is often used to study the phylogenetic relationships between diverse species because of its short length and maternal inheritance (Miya et al. 2003; Pardo et al. 2005). In addition, its gene structure, content, and sequence are conserved among vertebrates. Despite the value of mitochondrial DNA (mtDNA) in identifying similarities between organisms, there are only a few studies on *A. nadeshnyi*; Genbank contains only 13 sequences, including seven mitochondrial sequences of two *cox1*, two *cytb*, one 12S *rRNA*, and one *trnP*. Here, we determined the complete mitochondrial genome of *A. nadeshnyi* and determinedits phylogenetic position ofaccordingly. This mitogenome extends the available genetic data on *A. nadeshnyi* and enables the determination of its phylogenetic position.

## Materials

**Figure 1.**
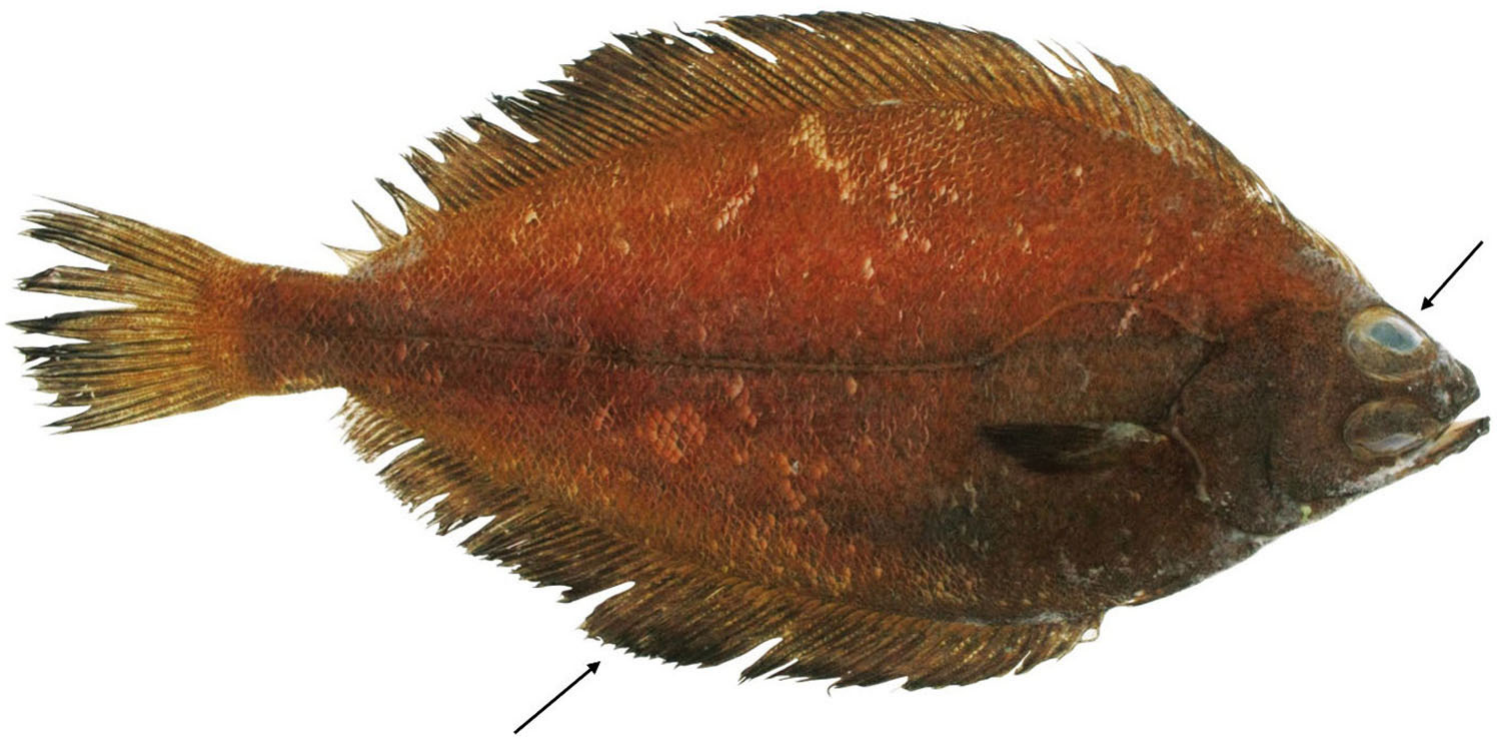
*Acanthopsetta nadeshnyi*. Fish fin was collected from this sample and used to determine the complete mitogenome of *A. nadeshnyi*. The photo was provided by Prof. Jinkoo Kim. The black arrows indicate characteristics features fins with black tips and scaly eyes.

A specimen was provided by the Marine Bio-Resource Information System (MBRIS; Voucher No. MRS002000103676; https://www.mbris.kr/pub/main/publicMainPage.do; jywoo1022@mabik.re.kr). Specimen was collected from Goseong, South Korea (34°90 ′N, 128°22 ′E) and deposited at the Pukyong National University storage facility (Figure 1).

## Methods

Genomic DNA was purified using a PureHelix™ *Genomic* DNA Prep Kit [Animals], Solution Type (NANOHELIX, Daejeon), and the *cox1* gene was amplified using a fish universal primer based on Ward et al. (2005). The amplicon sequence was analyzed by Macrogen (Seoul), and BLASTN searches against every nucleotide sequence in GenBank (Johnson et al. 2008) was conducted to identify the sample species. In addition, p-distance analysis was performed using Mega software version 11 (Tamura et al. 2021) with 1000 bootstrap replicates using the three most similar *cox1* sequences from the BLASTN results.

Library preparation was performed with an MGIEasy DNA Library Prep Set (MGI, Shenzhen) according to the manufacturer’s instructions. Raw data were generated by next-generation sequencing (NGS) using an MGISEQ-2000 platform, and deposited in the SRA database (Accession No. SRR22019788). The adapter was trimmed using Cutadapt version 4.1 (Martin 2011), and contig assembly was performed using CLC Genomics Workbench version 20.04 (QIAGEN, Hilden) using the default options in the *de novo* assembler. A circular sequence was mapped with the trimmed data sets using Geneious Software version 20.2.2 (https://www.geneious.com) to verify the complete mtDNA sequence of *A. nadeshnyi*. Tandem repeats and direct repeat motifs were predicted using Geneious software. Annotation was conducted on the MITOS WebServer (Bernt et al. 2013) and manually corrected using SnapGene software version 5.3.2 (GSL Biotech LLC, https://snapgene.com). The final mtDNA sequence was registered in GenBank (Accession No. OP028121). A mitogenome map was prepared using the CGView Server (Grant and Stothard 2008).

A maximum likelihood (ML) phylogenetic tree was constructed using MEGA software version 11 (Tamura et al. 2021) using mitogenomes from all 18 members of the family Pleuronectidae in GenBank and one member of the family Acipenseridae, *Acipenser dabryanus* (Accession No. AY510085), was used as the outgroup. All mitogenome sequences (Table 1) were collected from GenBank. Nucleotide sequences of protein-coding genes (PCGs) were gathered from each mitogenome sequence and aligned using the ClustalW multiple alignment tool in BioEdit with default options. Phylogenetic analysis was conducted using the GTR + G + I model with 1000 bootstrap replicates.

**Table 1.** Mitogenome sequence used in this study.

Scientific name	Accession number	Reference
*Acipenser dabryanus*	AY510085	Liu et al. 2017
*Acanthopsetta nadeshnyi*	OP02812	Described in this study
*Cleisthenes herzensteini*	KT223828	Bo et al. 2016
*Glyptocephalus stelleri*	MT258402	Kim and Jang 2022
*Hippoglossoides platessoides*	MN122825	Mjelle et al. 2008
*Hippoglossus hippoglossus*	CM020214	Mjelle et al. 2008
*Hippoglossus stenolepis*	AM749126	Mjelle et al. 2008
*Reinhardtius hippoglossoides*	AM749130	Mjelle et al. 2008
*Kareius bicoloratus*	AP002951	Miya et al. 2001
*Platichthys stellatus*	EF424428	Shi et al. 2013
*Pleuronichthys cornutus*	JQ639071	Shi et al. 2013
*Limanda aspera*	KP013094	Song et al. 2017
*Pleuronichthys japonicus*	KY038655	Song et al. 2017
*Pseudopleuronectes herzensteini*	ON127848	Chae et al. 2022
*Limanda limanda*	MN122886	Zheng et al. 2016
*Parophrys vetulus*	OL806591	Zheng et al. 2016
*Pseudopleuronectes yokohamae*	KT878309	Zheng et al. 2016
*Verasper moseri*	EF025506	He et al. 2008
*Verasper variegatus*	MK210571	Lim et al. 2019

## Results

BLASTN of *cox1* obtained by PCR showed an identity match of 99.85% with that of *A. nadeshnyi* (Accession No. MH032397), 95.98% with that of *Dexistes rikuzenius* (Accession No. MH032411), and 95.09% with that of *Hippoglossoides elassodon* (Accession No. JQ354126). The p-distance were 1.35 (*A. nadeshnyi*), 5.29 (*D. rikuzenius*), and 5.84 (*H. elassodon*), reflecting the same pattern as the BLASTN results. These results confirm that our flatfish specimen is *A. nadeshnyi*.

**Figure 2.**
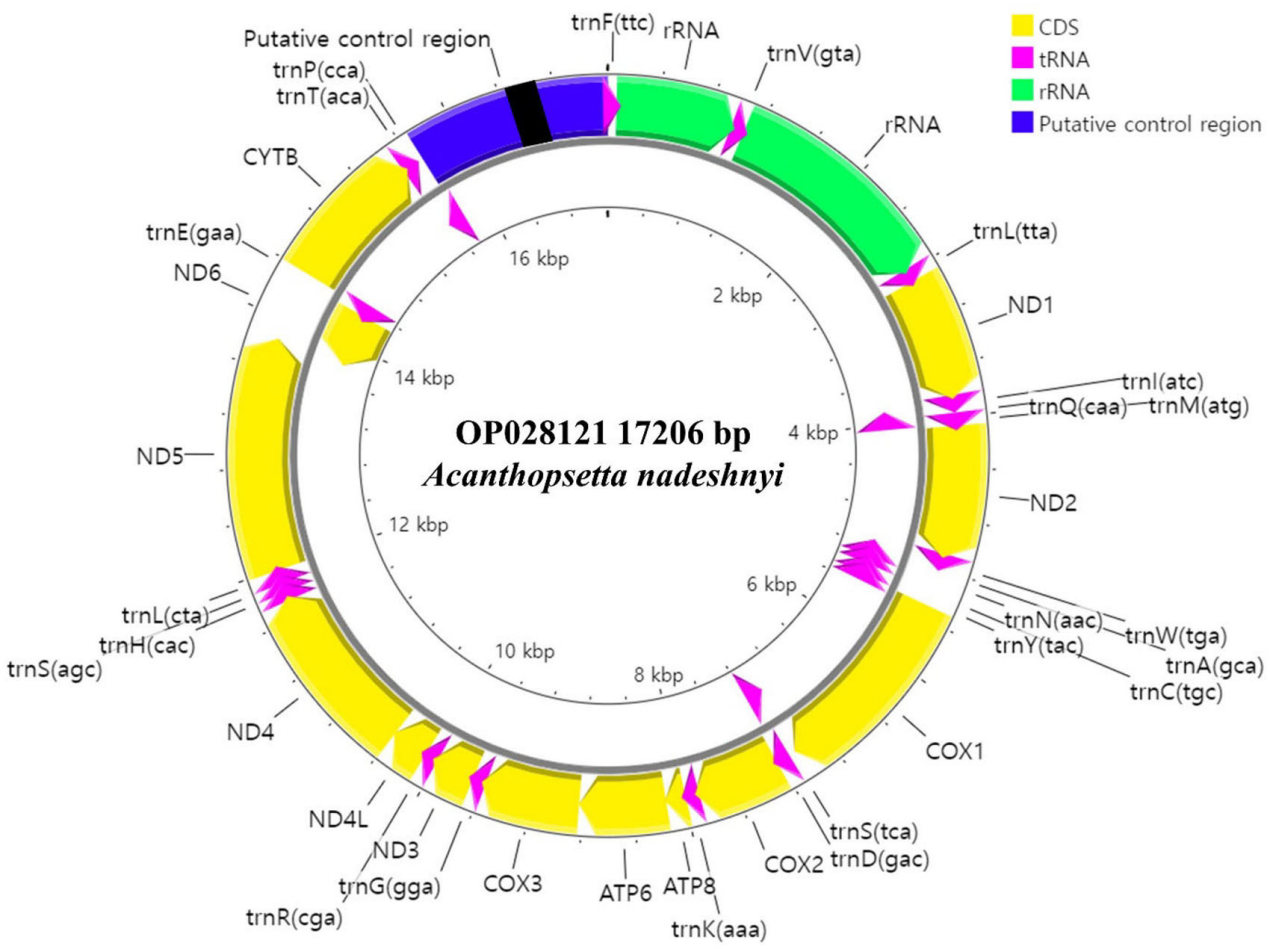
Complete mitochondrial genome map of *Acanthopsetta nadeshnyi*. The map comprised 37 genes, including 13 protein-coding genes, 22 transfer RNA genes, and two ribosomal RNA genes. The arrowheads indicate the direction of transcription. Highly similar 58–74 bp direct repeat motifs occur in the putative control region (black box).

In total, 20,481,617 read-pairs were produced by NGS, from which the mitogenome of *A. nadeshnyi* (17,206 bp) was assembled with a mean coverage of 513.0 ± 97.9 (Figure S1). The mitogenome encoded 37 genes, including 13 PCGs, 22 transfer RNA (tRNA) genes, and two ribosomal RNA (rRNA) genes (Figure 2). Every PCG was encoded on the positive strand except *nad6*, which was encoded on the negative strand. ATG was a start codon in 12 PCGs (*nad1*, *nad2*, *cox2*, *atp8*, *atp6*, *cox3*, *nad3*, *nad4l*, *nad4*, *nad5*, *nad6*, and *cob*), whereas only *cox1* started with the GTG codon. Transcription was stoped with the TAA codon in six genes (*nad1*, *atp8*, *atp6*, *nad4l*, and *nad5*, *nad6*). In contrast, *nad2*, *cox2*, *nad3*, *nad4*, and *cob* stoped with truncated T-, and *cox3* terminated with truncated TA-.

Including two *trnL* and two *trnS*, the mitogenome of *A. nadeshnyi* comprised 22 tRNA genes 14 tRNA genes were encoded on the positive strand and the others were encoded on the negative strand (Figure 2).

The small rRNA gene was 949 bases long and located between *trnF* and *trnV*. The large rRNA was 1714 bases long and is located between *trnV* and *trnL(tta)*. The putative control region was 1528 bases long and located between *trnP* and *trnF*. Conserved sequence box D (5′- CCTGGCATTTGGTTCC-3′), a pyrimidine sequence run (5′- TTCTCTTTTTTTTTTTCCTTTC-3′), and two conserved sequence boxes (CSB-2: 5′-AAACCCCCCTACCCCCC-3′; CSB-3: 5′-TGAAAACCCCCCGGAAACA-3′) occurred in the putative control region. There were no tandem repeats, but highly similar 58–74 bp direct repeat motifs were found at positions 802–1152 of the putative control region. In addition, the OL region was observed in another non-coding region that may fold into a stem-loop secondary structure.

**Figure 3.**
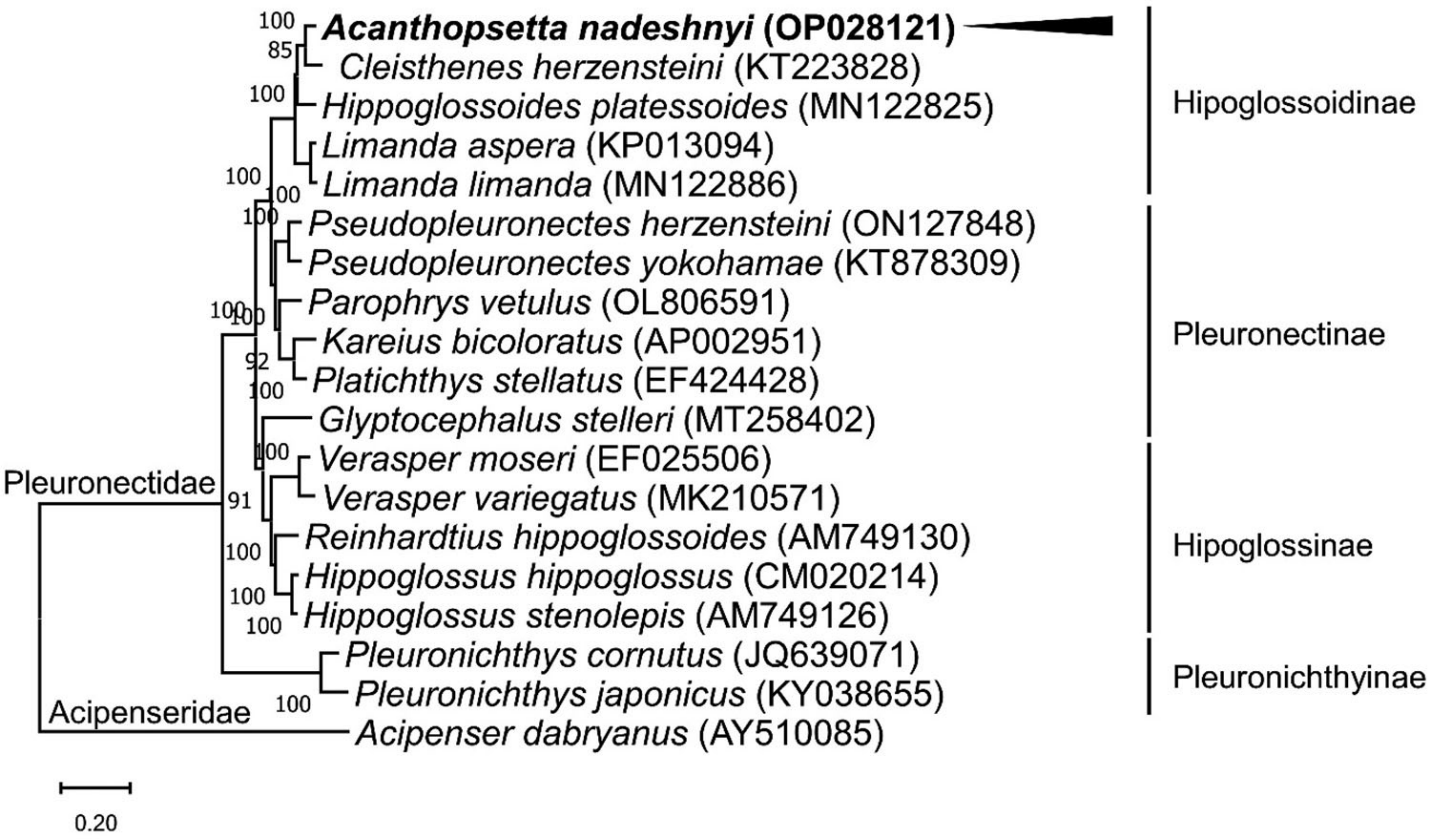
Phylogenetic tree of *Acanthopsetta nadeshnyi* and related species. Based on the maximum likelihood (ML) tree, the phylogenetic position of *A. nadeshnyi* was analyzed using 13 PCGs from the mitogenomes of 18 members of the Pleuronectidae family with *Acipenser dabryanus* (Accession No. AY510085) as an outgroup. GenBank accession numbers for the mitogenome sequences are provided next to the species names. The node numbers correspond to the posterior probabilities of Bayesian inference. The black arrow and bold fonts highlight the mitogenome of *A. nadeshnyi.*

The ML phylogenetic tree of the family Pleuronectidae showed that *A. nadeshnyi* was grouped with *Cleisthenes herzensteini* (Figure 3). The mitogenome of *A. nadeshnyi* was separated into different nodes, including species of the subfamily Hippoglossoidinae.

## Discussion and conclusion

Here, we reported that the complete mtDNA sequence of *A. nadeshnyi* had a comparable gene composition with that generally observed in vertebrate mtDNA (Kolesnikov and Gerasimov 2012). The composition and gene order of this mitogenome matched those of teleosts, reflecting the limited occurrence of mitogenome rearrangements in fish (Gong et al. 2013). In the Pleuronectidae family, various tandem repeats contribute to the relatively large size of the control regions (Zheng et al. 2016; Song et al. 2017). However, no tandem repeats were found in the mitogenome of *A. nadeshnyi* similar to that for *G. stelleri* (Kim and Jang 2022), whereas highly similar direct repeat motifs and several conventional motifs were observed. These highly similar direct repeat motifs can potentially extend the size of the control region. This suggests that tandem repeats and highly similar direct repeat motifs may be useful biomarkers for identifying relationships between Pleuronectidae species. Considering the limited number of studies on *A. nadeshnyi*, the present study contributes new genetic information for Pleuronectidae.

## Supplementary Material

Supplemental Material

Supplemental Material

## Data Availability

The genome sequence data supporting this study’s findings are available in GenBank of NCBI at (https://www.ncbi.nlm.nih.gov/) under accession no. OP028121. The associated BioProject, SRA, and Bio-Sample numbers are PRJNA862354, SRR22019788, and SAMN29974750, respectively.
