# Supplementary material for: The study on the complete mitochondrial genome of Acanthopsetta nadeshnyi and its phylogenetic position
Source: Mitochondrial DNA B Resour. 2023 Aug 10;8(8):852–6. doi: 10.1080/23802359.2023.2241670 (PMC10424613; doi:10.1080/23802359.2023.2241670)
Supplement: Supplemental Material [file TMDN_A_2241670_SM1582.docx]

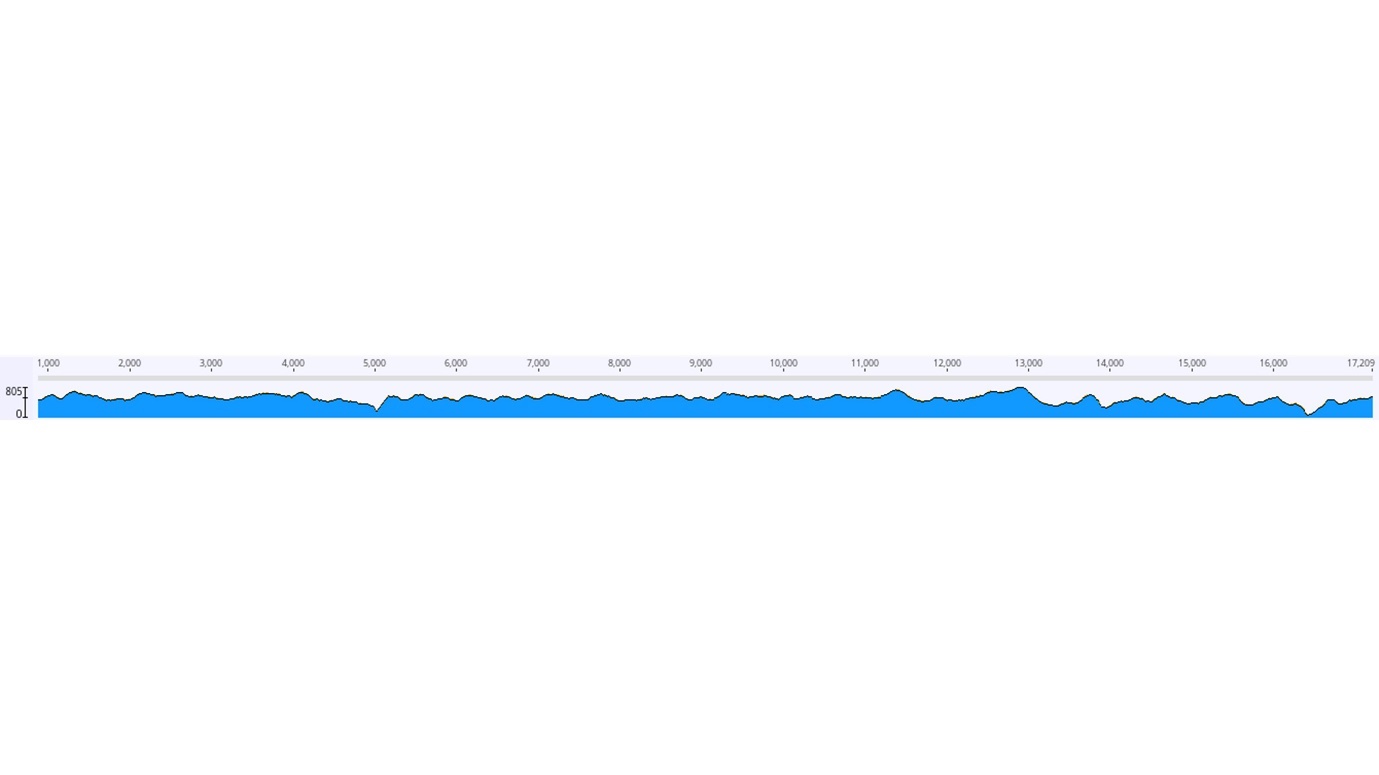


Supplementary Figure S1. Read mapping depth of the mitogenome sequence

The read mapping depth is represented by the blue-scale bar. The x-axis and y-axis indicate nucleotide position and coverage, respectively. The maximum, average, and minimum coverage are 805x, 513x, and 65x, respectively.
