# Supplementary material for: The study on the complete mitochondrial genome of Acanthopsetta nadeshnyi and its phylogenetic position
Source: Mitochondrial DNA B Resour. 2023 Aug 10;8(8):852–6. doi: 10.1080/23802359.2023.2241670 (PMC10424613; doi:10.1080/23802359.2023.2241670)
Supplement: Supplemental Material [file TMDN_A_2241670_SM1574.pdf]

Editing Certificate

This document certifies that the manuscript listed below has been edited to ensure language and grammar accuracy and is error free in these aspects. The logical presentation of ideas and the structure of the paper were also checked during the editing process. The edit was performed by professional editors at Editage, a brand of Cactus Communications. The author's core research ideas were not altered in any way during the editing process. The quality of the edit has been guaranteed, with the assumption that our suggested changes have been accepted and the text has not been further altered without the knowledge of our editors.

MANUSCRIPT TITLE

Study on the complete mitochondrial genome of Acanthopsetta nadeshnyi and its phylogenetic position

AUTHORS

Jun Young Chae

ISSUED ON

July 15, 2023

JOB CODE

OIAQD_1

THE EDITAGE Promise QUALITY GUARANTEE

Vikas Narang

Vikas Narang
Chief Operating Officer - Editage

editage helping you get published

Since 2002, Editage has helped over 430,000 authors publish around 1.2 million research papers in scholarly journals across over 1000 disciplines through editorial, translation, transcription, and publication support services. Editage is a brand of Cactus Communications (cactusglobal.com), a science communication and technology company.

CACTUS.

GLOBAL : +1(833) 979-0061 | request@editage.com

KOREA : 02-3478-4396 | submit-korea@editage.com

editage.com | editage.co.kr | editage.jp | editage.cn | editage.com.br | editage.com.tw | editage.de
